# Fibrinogen binding is affected by amino acid substitutions in C-terminal repeat region of fibronectin binding protein A

**DOI:** 10.1038/s41598-019-48031-5

**Published:** 2019-08-12

**Authors:** Nadia N. Casillas-Ituarte, Alex C. DiBartola, Megan J. Broughton, Lumarie Pérez-Guzmán, Robert M. Wheeler, Makoto Ibaraki, B. Alexis Lower, James A. Dunn, Brian H. Lower, Vance G. Fowler, Magnus Hӧӧk, Lauren M. McIntyre, Steven K. Lower, Batu K. Sharma-Kuinkel

**Affiliations:** 1 Ohio State University, Columbus, Ohio, 43210 USA; 2 Duke University Medical Center, Durham, North Carolina 27708 USA; 3 Texas A&M Health Science Center, Houston, Texas 77030 USA; 4 University of Florida, Gainesville, Florida 32610 USA

**Keywords:** Pathogens, Molecular medicine

## Abstract

Fibronectin-binding protein A (FnBPA), a protein displayed on the outer surface of *Staphylococcus aureus*, has a structured A-domain that binds fibrinogen (Fg) and a disordered repeat-region that binds fibronectin (Fn). Amino acid substitutions in Fn-binding repeats (FnBRs) have previously been linked to cardiovascular infection in humans. Here we used microtiter and atomic force microscopy (AFM) to investigate adhesion by variants of full-length FnBPA covalently anchored in the outer cell wall of *Lactococcus lactis*, a Gram-positive surrogate that otherwise lacks adhesins to mammalian ligands. Fn adhesion increased in five of seven FnBPA variants under static conditions. The bond targeting Fn increased its strength with load under mechanical dissociation. Substitutions extended bond lifetime (1/*k*_*off*_) up to 2.1 times for FnBPA-Fn. Weaker adhesion was observed for Fg in all FnBPA variants tested with microtiter. However, mechanical dissociation with AFM showed significantly increased tensile strength for Fg interacting with the E652D/H782Q variant. This is consistent with a force-induced mechanism and suggests that the dock, lock, and latch (DLL) mechanism is favored for Fg-binding under mechanical stress. Collectively, these experiments reveal that FnBPA exhibits bimodal, ligand-dependent adhesive behavior. Amino acid substitutions in the repeat-region of FnBPA impact binding to both ligands. This was unexpected for Fg since all variants have the same A-domain sequence, and the Fg-binding site is distant from the repeat region. This indicates that FnBRs may fold back on the A-domain in a way that impacts the DLL binding mechanism for Fg.

## Introduction

*Staphylococcus aureus* is an opportunistic pathogen that can cause a wide range of afflictions from superficial and self-limiting infections to more serious endovascular infections, such as infective endocarditis^1–3^. These infections are facilitated by cell-wall anchored adhesins of the MSCRAMM (microbial surface components recognizing adhesive matrix molecules) type that bind to host blood proteins, including fibronectin (Fn) and fibrinogen (Fg). Fg is present in the bloodstream at a concentration of 1.5–4.5 g/L^4,5^; while Fn concentration is 0.2–0.4 g/L^6^. Fg plays a role in blood clotting whereas Fn plays a role in cell migration during wound healing^4,7^. Fn and Fg are also found in the extracellular matrix, damaged tissue, and coatings on implanted devices^2,8^.

**Figure 1:**
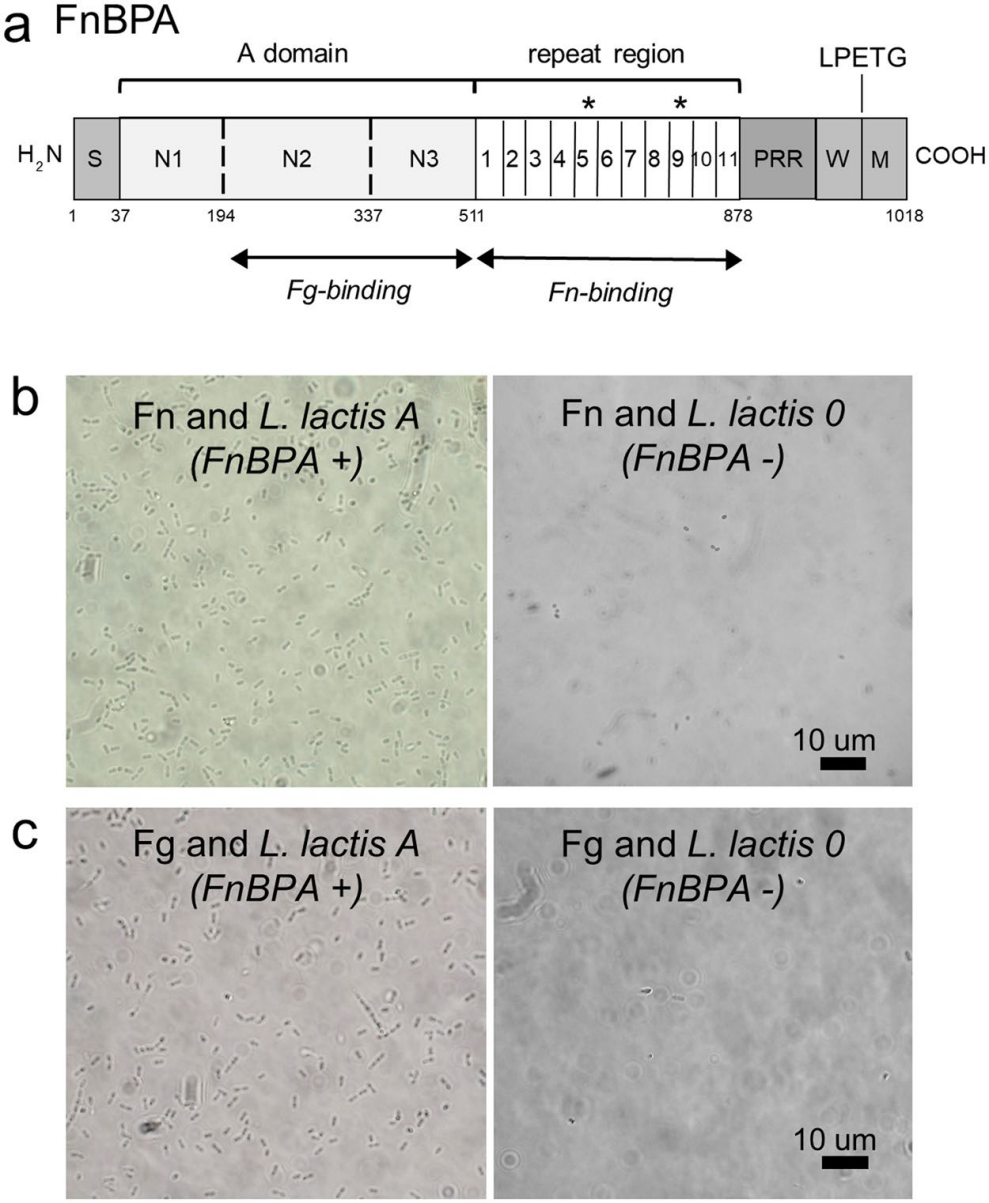
(**a**) Schematic representation of fibronectin binding protein A (FnBPA) from *S*. *aureus* 8325-4 (not to scale). The N-terminus contains a signal sequence (S) followed by the A domain, which contains the N2N3 binding site for fibrinogen (Fg). Adjacent to the A domain are eleven repeats that bind to fibronectin (Fn). The C-terminus contains a proline-rich repeat (PRR), wall (W) and membrane (M) spanning domains. Numbers across the bottom correspond to amino acid residues. The asterisks above the boxes indicate the repeat regions with reported amino acid substitutions (at positions 652, 782 and 786). Optical microscopy images of *Lactococcus lactis* expressing (*L*. *lactis* A, FnBPA^+^) or not expressing (*L*. *lactis* 0, FnBPA^−^) cell wall associated FnBPA following incubation on (**b**) Fn-coated glass slides and (**c**) Fg-coated glass slides. Slides were coated with 100 µg/mL of Fn or Fg.

A critical cell-wall anchored adhesin implicated in *S*. *aureus* infections is fibronectin-binding protein A (FnBPA)^9^. This bacterial protein contains an N-terminal A-domain that binds to Fg, and includes subdomains N1, N2, and N3, followed by the Fn-binding repeats (FnBRs), and the C-terminal cell-wall associated domain (Fig. 1a). The ligand-binding sites in FnBPA differ in that the A domain is a structured region whereas the FnBR region consists of 11 non-identical, unfolded, repeats. In terms of binding mechanisms, FnBPA binds Fg through a variant of the “dock, lock, and latch” (DLL) mechanism, which is initiated by docking of the C-terminus of the Fg γ-chain into a trench between N2/N3 subdomains^1,10,11^. For Fn binding, the FnBRs of FnBPA bind through a tandem β-zipper mechanism by forming anti-parallel β-strand along the type-I modules at the N-terminus of Fn^12,13^.

Previous clinical studies have identified nonsynonymous, single nucleotide polymorphisms in the *fnbA* gene associated with cardiovascular infections in human patients^14–16^. Specifically, these prior studies found amino acid substitutions in FnBR-5 and FnBR-9 of FnBPA in *S*. *aureus* isolated from patients with infected cardiac implants and infective endocarditis. These variations confer a higher binding affinity for Fn in *S*. *aureus*^17^ suggesting that strains with these substitutions are more likely to evade host defenses and/or colonize damaged tissue or implanted devices, where host proteins including Fn have been recruited as part of the body’s healing process.

In this study, we examined three amino acid substitutions in FnBRs that were associated with disease (E652D, H782Q, K786N; see locations in Fig. 1a) to determine their impact, if any, on binding to Fn when expressed in *Lactococcus lactis*. *L*. *lactis* is an ideal surrogate strain because it is gram positive, like *S*. *aureus*, but lacks adhesins for mammalian proteins including Fg and Fn^9,18,19^. We also tested adhesion of *L*. *lactis* to Fg since this ligand plays a key role in healing and is present as coatings on implants^8^. A microtiter assay and a more advanced atomic force microscopy (AFM) technique were used to evaluate binding between Fn or Fg and each of seven FnBPA variants with amino acid substitutions in FnBR-5 (E652D) and/or FnBR-9 (H782Q, K786N). These two techniques allow us to evaluate the mechanical responses of the ligand-receptor pairs in the absence (microtiter) and presence (AFM) of physical stress. This condition becomes relevant since surface-attached bacteria, including *S*. *aureus* are often subjected to physical stresses in the human body^20^. Additionally, other *S*. *aureus* adhesins (*i*.*e*., clumping factor A and B, ClfA and ClfB, respectively) have shown force-enhanced adhesion when subjected to mechanical stress^21–23^.

Overall, the results presented herein show that amino acid substitutions in the repeat region of FnBPA previously associated with infection in humans do indeed impact the strength of binding with the blood host proteins, Fn and Fg. Specifically, these substitutions in the repeat region of FnBPA enhance binding to Fn and conform to a Bell-type model describing a single dissociation pathway^24,25^. More surprising was the observed impact of these substitutions on Fg binding. While the A-domain, (*i*.*e*., the known location of the Fg binding site) was identical for all FnBPA variants, substitutions in the repeat region altered the strength of the Fg interaction. This indicates that the unstructured FnBRs interact with Fg, possibly folding back on the A-domain, in a way that impacts the DLL mechanism. Perhaps, more importantly, we found that some substitutions in FnBPA significantly strengthened interactions with Fg upon application of a tensile force to the bond. This is the first evidence of a force-sensitive mechanism for FnBPA-Fg observed in experiments with full-length proteins. Based on the results presented herein, it appears that interactions of FnBPA with Fg molecules (more than Fn) are susceptible to conformation changes under mechanical stress that impact binding.

## Results

### FnBPA construction and surface expression

Both Staphylococci and Lactococci process cell wall proteins in a similar fashion, using the conserved LPXTG C-terminal motif to covalently anchor proteins into the cell wall envelope^26^. This process includes an effective sortase signaling mechanism that requires the conserved N-terminal signal peptide and a C-terminal motif sorting signal. Staphylococcal MSRAMMS adhesins, including FnBPA are cell-wall anchored proteins that follow this mechanism to be expressed on the outer envelop of the bacteria surface^18,19^.

Full-length FnBPA expression on *L*. *lactis* was accomplished by using the previously described *E*. *coli-Lactococci* shuttle expression vector pOri23^18,19^ (see supplementary information for details). The *fnbA* gene of *S*. *aureus* 8325–4 was used as the template. Single, double or triple mutations on *fnbA* were created with site directed mutagenesis (Table 1, Supplementary Fig. S1). The desired mutations in *fnbA* gene were verified by sequencing the plasmids in all seven combinations of E652D, H782Q, and K786N (Supplementary Fig. S1). The resulting pOri23-fnbA plasmids with mutation(s) on *fnbA* were transformed into *L*. *lactis subsp*. *cremoris* 1363 according to published methods^18,19^.

**Table 1:** Bacteria strains used in this study.

*L*. *lactis* name	Plasmid expression vector & mutations	Analysis^a^
*L*. *lactis* 0	pIL253 (negative control)	MT, AFM
*L*. *lactis* A	pOri23 + *fnbA* (reference *fnbA*)	MT, AFM
*L*. *Lactis E652D*^*s*^	pOri23 + *fnbA*E652D (GAG to GAT)	MT
*L*. *Lactis H782Q*^*s*^	pOri23 + *fnbA*H782Q (CAT to CAA)	MT
*L*. *Lactis K786N*^*s*^	pOri23 + *fnbA*K786N (AAA to AAT)	MT
*L*. *Lactis E652D/H782Q*^*d*^	pOri23 + *fnbA*E652D/H782Q	MT, AFM
*L*. *Lactis E652D/K786N*^*d*^	pOri23 + *fnbA*E652D/K786N	MT
*L*. *Lactis* H782Q/K786N^*d*^	pOri23 + *fnbA*H782Q/K786N	MT
*L*. *Lactis* E652D/H782Q/K786N^*t*^	pOri23 + *fnbA*E652D/H782Q/K786N	MT, AFM

^*a*^Type of analysis: MT = microtiter, AFM = atomic force microscopy. ^*s*^Single, ^*d*^double, or ^*t*^triple amino acid polymorphism(s) in *fnbA*.

Overlapping growth curves were observed for the wild-type and the seven FnBPA variants (Supplementary Fig. S2). Similar rate and extent of growth suggest comparable physiological states for all strains. Flow cytometry studies (Supplementary Fig. S3) showed similar peak area ratios of the cell population expressing FnBPA and unstained control, while Western Blots (Supplementary Fig. S4) also revealed similar band intensity for the *fnbA* mutants and wild type confirming that *L*. *lactis* A and its seven variants expressed FnBPA at approximately equivalent concentrations.

### Static microtiter assay of adhesion to ligands

**Figure 2:**
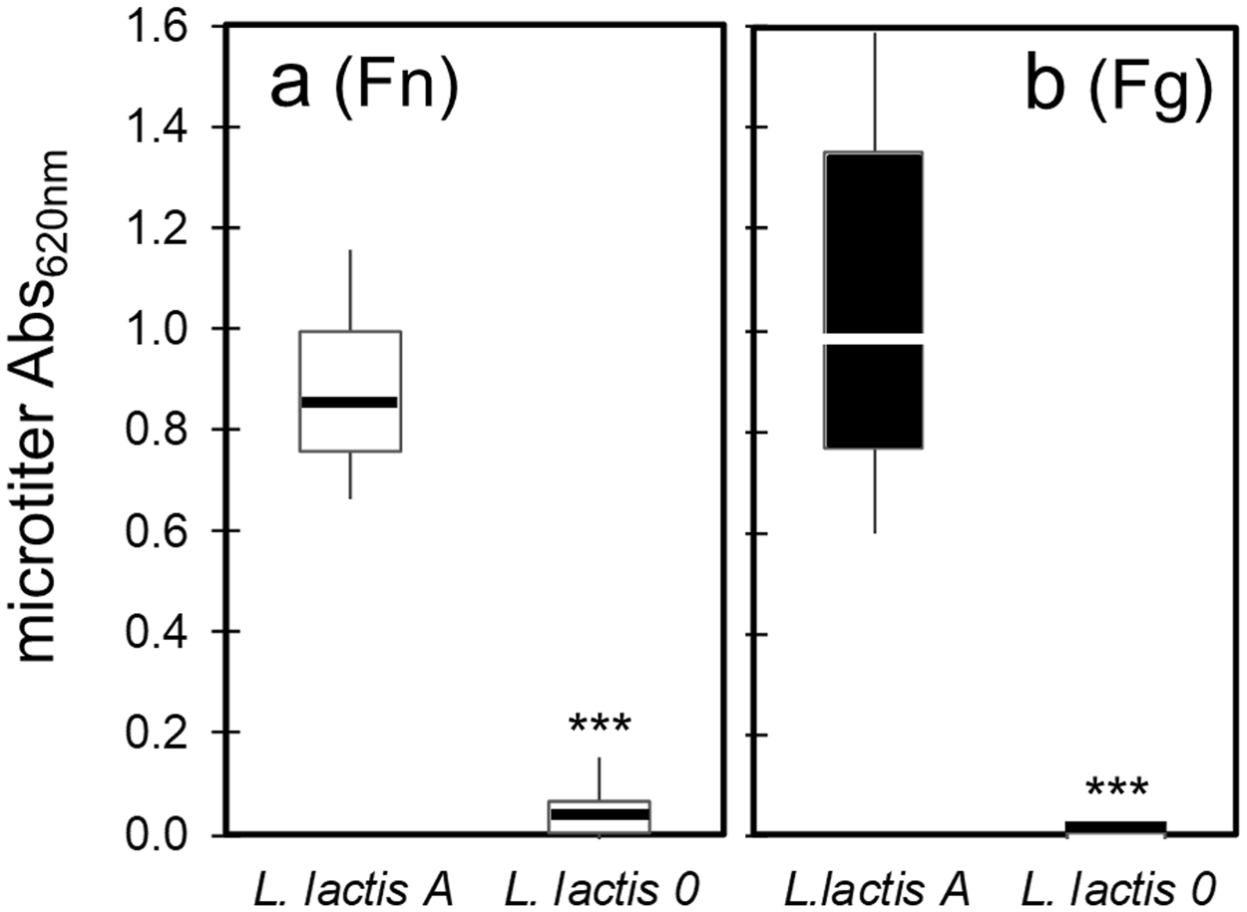
Box and whisker plots of *L*. *lactis* A or 0 binding to (**a**) Fn-coated polystyrene and (**b**) Fg-coated polystyrene, as determined by absorbance in microtiter wells. Whisker ends 9^th^ and 91^st^ percentiles. Box ends 25^th^ and 75^th^ percentiles. Median is marked with a horizontal line. *p*-values calculated using Mann-Whitney where *p* < 0.0001 is indicated by ***. Microtiter wells were coated with protein by incubating plates in 100 µg/mL of Fn or Fg.

Functional FnBPA expression on *L*. *lactis* was confirmed by testing adhesion to Fn- or Fg-coated glass slides. *L*. *lactis* A adhered strongly to Fn and Fg-coated substrates compared to minimal adhesion observed with *L*. *lactis* 0 (Fig. 1b,c). Adhesion of these two strains was further tested using the microtiter assay (Fig. 2). Expression of *fnbA* in *L*. *lactis* A resulted in statistically significant (*p* < 0.0001) enhancement in adhesion to both ligands; from 0.017 to 0.887 for Fn, and from 0.007 to 1.055 for Fg. The microtiter adhesion assay showed greater number of *L*. *lactis* A cells on the Fg-coated plates compared to Fn (Fig. 2; *p* = *0*.*0003*). This observation is assigned to more Fg molecules being present on the plates. The same concentration of both ligands (100 µg/ml) was used to coat the plates, however Fn and Fg have different molecular weights (*i*.*e*., Fn is ~440 kDa; Fg is ~340 kDa). The estimated Fg/Fn ratio is 1.3 whereas the ratio of the average absorbance is 1.2 (see Supplementary Information for details).

**Figure 3:**
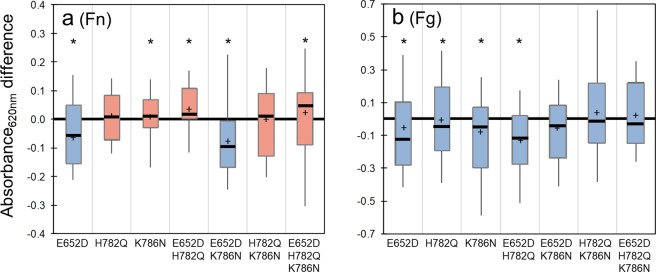
Box and whisker plots of *L*. *lactis* strains binding to (**a**) Fn-coated polystyrene and (**b**) Fg-coated polystyrene microtiter wells, as determined by the difference of variant absorbance minus the absorbance of the wild-type *L*. *lactis* A. Whisker ends 9^th^ and 91^st^ percentiles. Box ends 25^th^ and 75^th^ percentiles. Median is marked with horizontal line and average with cross. Blue- or red-colored boxes represent median absorbance differences that are, respectively, weaker or stronger than the absorbance of *L*. *lactis* A (the wild type strain). For example, a negative value (blue color box) indicates the *L*. *lactis A* wild-type cells adhered in greater number than a particular variant. *p*-values calculated using Mann-Whitney where *p* < 0.05 is indicated by *. Microtiter wells were coated with protein by incubating plates in 100 µg/mL of Fn or Fg.

The microtiter assay was then used to examine the adhesion of each of the seven FnBPA variants with amino acid substitutions that have been previously associated with infections of cardiac implants in human patients. More than 20 different microtiter plates were used for these experiments. To reduce the variability in absorbance readings among different plates, we determined the relative difference in absorbance for each variant minus absorbance of *L*. *lactis* A, a strain included in all plates. Results are shown as box-and-whisker plots (Fig. 3) to reveal the distribution of values in each data set. Blue- or red-colored boxes represent a variant whose median value of adhesion is weaker or stronger, respectively, than *L*. *lactis* A.

Median Fn adhesion was enhanced above that of *L*. *lactis* A in five of the seven FnBPA variants (Fig. 3a, Table 2). The “enhanced” variants included H782Q, K786N, E652D/H782Q, H782Q/K786N, and E652D/H782Q/K786N. The E652D, and E652D/K786N variants presented a decrease in adhesion when compared to *L*. *lactis* A. By contrast, adhesion to Fg was diminished in all seven FnBPA variants when compared to the wild-type (Fig. 3b; Table 2).

**Table 2:** Median Abs_620nm_ values for *L*. *lactis* strains binding to Fn-coated polystyrene and Fg-coated wells as determined by the crystal violet microtiter assay.

Substitutions in FnBPA		E652D	H782Q	K786N	E652D H782Q	E652D K786N	H782Q K786N	E652D H782Q K786N
Fn	Median	−*0.055*	**0.002**	**0.012**	**0.026**	−*0.096*	**0.005**	**0.057**
	*p* values	0.030*	0.193	0.003*	0.001*	<0.0001*	0.623	0.002*
Fg	Median	−*0.122*	−*0.056*	−*0.058*	−*0.117*	−*0.046*	−*0.009*	−*0.040*
	*p* values	0.0002*	0.022*	0.005*	<0.0001*	0.335	0.752	0.096

Bold text indicates higher than wild and italic represents lower than *L*. *lactis* expressing wild-type sequence of *fnbA*. P-values calculated using Mann-Whitney test relative to *L*. *lactis* expressing wild-type *fnbA* where *p* < 0.05 is indicated by *.

Based on the microtiter assay results, four strains were selected for further analysis with AFM. *L*. *lactis* A was selected as the baseline and *L*. *lactis* 0 as the negative control. The E652D/H782Q variant was selected because it stood out with statistically enhanced adherence towards Fn but diminished adherence to Fg (see Fig. 3). The “triple mutant” E652D/H782Q/K786N was also analyzed with AFM since a greater number of polymorphisms is expected to cause greater impact on binding under dynamic conditions.

### Dynamic force measurement of ligand binding

AFM differs from the microtiter assay in several key ways. AFM directly measures bond strength through a dynamic process of pushing and pulling on ligand-receptor linkages at nonequilibrium. Furthermore, AFM is performed on living bacteria.

**Figure 4:**
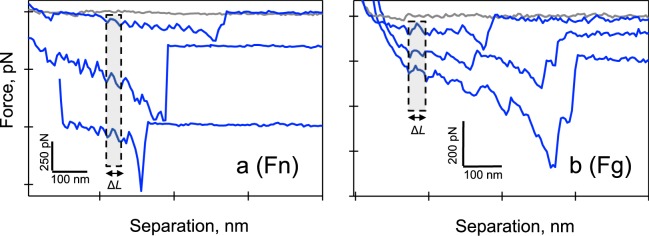
Force spectra showing specific interactions (in blue) between full-length FnBPA expressed on the cell wall of *L*. *lactis* and (**a**) full-length Fn, and (**b**) full-length Fg. Spectra from nonspecific interactions (in gray) between the cell wall of *L*. *lactis* 0 (no FnBPA, empty vector) and the two ligands are also shown. Peak-to-peak distance (ΔL) for Fn is 30 ± 4 nm and 36 ± 10 nm for Fg. AFM tips were coated with protein by incubating tips in 100 µg/mL of Fn or Fg prior to force measurements.

With AFM, nonspecific binding interactions were observed between *L*. *lactis* 0 and the ligands (Fig. 4a,b, gray curves). In contrast, specific interactions (Fig. 4a,b, blue curves) often presented successive binding events with a peak-to-peak distance (ΔL) of 30 ± 4 nm and 36 ± 10 nm, for Fn and Fg, respectively. These ΔL values are in the range of the unfolding distance of multiple Fn repeats (*i*.*e*., 28 nm) and the forced unfolding of Fg structural elements including the α-helical coils and the globular elements in the γ-nodules (*i*.*e*., ~31 nm)^27–29^. These findings confirm the specificity of the AFM measurement for FnBPA molecules on *L*. *lactis*. Furthermore, these ΔL values were used as a control to verify the presence of the host proteins on the AFM tip while interacting with *L*. *lactis* expressing FnBPA (see Methods section for details).

**Figure 5:**
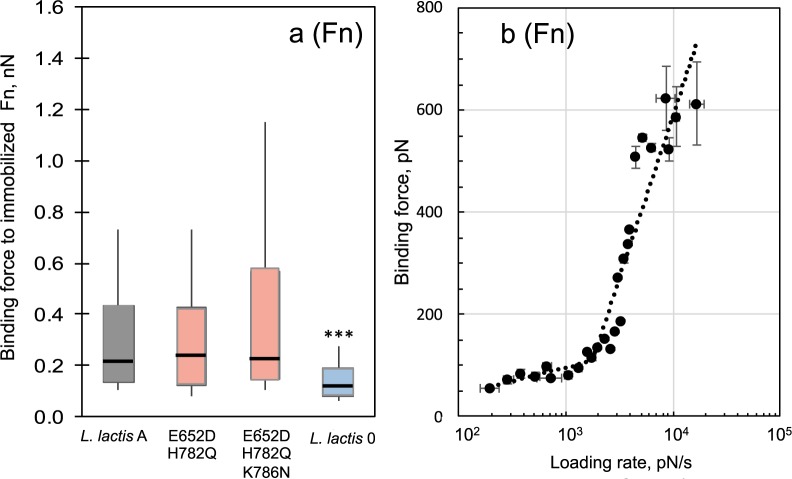
(**a**) Box and whisker plots of *L*. *lactis* strains binding to Fn as determined by AFM. Whisker ends 9^th^ and 91^st^ percentiles. Box ends 25^th^ and 75^th^ percentiles. Median is marked with horizontal line. Blue- or red-colored boxes represent median force values that are, respectively, less than or greater than the wild type strain (*L*. *lactis* A). *P*-values calculated using Mann-Whitney, where *p* < 0.001 is indicated by ***. (**b**) Dependence of binding or dissociation force (*f*; pN) on the logarithm of the loading rate, (*r*; pN s^−1^) between FnBPA expressed on the surface of the *L*. *lactis* E652D/H782Q and Fn. This dependence is described by the Bell model *f* = (*k*_*B*_*T/x*_*β*_) × (*In* (*r x*_*β*_*/k*_*off*_ *k*_*B*_ *T*)), where *r*(pNs^−1^) is the product of the retraction velocity (nm s^−1^) and the slope of the force-distance curve at binding (pN nm^−1^), *k*_*off*_ is the dissociation rate constant in the absence of the applied force, *x*_*β*_ is the position of the activation barrier, and *k*_*B*_*T* is the thermal energy where *k*_*B*_ is Boltzmann constant and *T* is temperature (4.1 pN nm at room temperature). AFM measurements were conducted in PBS at retraction velocities between 0.05 and 18.8 µm/s. AFM tips were coated with protein by incubating tips in 100 µg/mL of Fn or Fg prior to force measurements.

Figure 5a are box-and-whisker plots summarizing the binding force of Fn from FnBPA as probed with AFM. With Fn, nonspecific interactions (*L*. *lactis* 0) were significantly weaker (*p* < 0.0001) than FnBPA-Fn interactions. Stronger bonds were observed for both the double and triple mutants relative to *L*. *lactis A*. Median binding forces were 1.05 to 1.10x stronger for E652D/H782Q and E652D/H782Q/K786N relative to *L*. *lactis A* (Supplementary Table S1). These results are similar to the microtiter assay, which showed higher adherence for both variants towards Fn.

For the Fn ligand, AFM was performed at different loading rates to evaluate the FnBPA-Fn interactions under different mechanical stress according to the Bell-Evans model^24,25^. From this model the unstressed, off-rate binding constant or dissociation rate constant (*k*_*off*_) was determined by plotting the binding force versus the loading rate (Fig. 5b). Two linear regimes were detected for all FnBPA-Fn complexes and were defined as energy barriers along the unbinding pathway of the system^25^. The dissociation rate constants from each barrier were calculated. For the lower-strength regime the *k*_*off*_ values were 3.7 ± 0.6, 1.4 ± 0.3, and 1.8 ± 0.1 sec^−1^ for *L*. *lactis* A, E652D/H782Q, and E652D/H782Q/K786N, respectively. In the higher-strength regime, dissociation rate constants were 3.4 ± 0.1, 4.0 ± 0.1, and 1.8 ± 0.1 sec^−1^ for *L*. *lactis* A, E652D/H782Q, and E652D/H782Q/K786N, respectively. Nonspecific binding forces between *L*. *lactis* 0 and Fn exhibited a single regime, which yielded the shortest bond lifetime estimate (1/*k*_*off*_ = 0.05 s).

**Figure 6:**
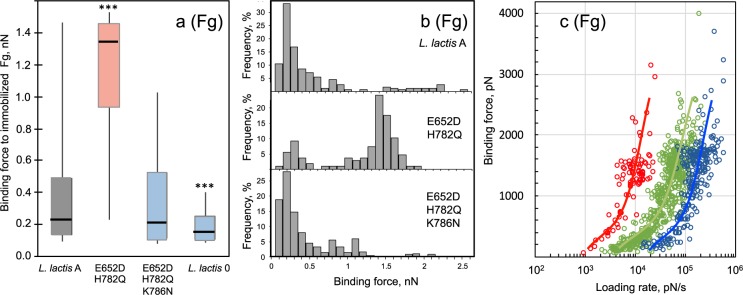
(**a**) Box and whisker plots of *L*. *lactis* strains binding to Fg as determined by AFM. Whisker ends 9^th^ and 91^st^ percentiles. Box ends 25^th^ and 75^th^ percentiles. Median is marked with horizontal line. Blue- or red-colored boxes represent median force values that are, respectively, less than or greater than the wild type strain (*L*. *lactis* A). *P*-values calculated using Mann-Whitney, where *p* < 0.001 is indicated by ***. (**b**) Histogram representation of the binding forces shown in panel (**a**). One group of binding forces at ~200 pN is observed in all of the strains. A second set of higher binding forces (~1.3 nN) is present only in the E652D/H782Q variant. (**c**) Dependen**c**e of binding or dissociation force (*f*; pN) on the logarithm of the loading rate, (*r*; pN s^−1^) between Fg and FnBPA expressed on the surface of the *L*. *lactis* E652D/H782Q. Different force-loading regimes (shown as different colors) are indicative of multiple binding pathways. AFM measurements were conducted in PBS at retraction velocities from 0.05 to 18.8 µm/s. AFM tips were coated with protein by incubating tips in 100 µg/mL of Fn or Fg prior to force measurements.

For Fg, Fig. 6a shows box-and-whisker plots summarizing the binding force for FnBPA. Nonspecific interactions were significantly weaker (*p* < 0.0001) than the FnBPA-Fg forces, similar to AFM observations for Fn. The median binding force for Fg:E652D/H782Q/K786N interactions was slightly weaker than the interactions with the *L*. *lactis* A reference, similar to results obtained with microtiter. A notable discrepancy for Fg, was the strength of interactions observed for the E652D/H782Q variant. The microtiter assay showed statistically weaker adhesion for E652D/H782Q, but AFM showed statistically stronger bond strength (*p* < 0.0001 vs. *L*. *lactis A*). A closer inspection of Fig. 6a shows a wide data distribution as seen by the ends of the whiskers for *L*. *lactis* A and the E652D/H782Q variant.

Plotting the FnBPA-Fg interactions as a frequency plot (see Fig. 6b) shows two modes of binding for this ligand. One group of binding forces, centers around 200 pN and it is present in all of the strains. A second set of forces centered at ~1.3 nN is present almost exclusively for the E652D/H782Q variant. This indicates two binding modes for this particular variant; one similar to that found for the wild and triple mutant (in ~25% of the FnBPA-Fg interactions), and a second mode of binding with remarkably strong forces (in ~75% of the interactions). That is, nanonewton binding forces were observed in the majority of Fg interaction for the E652D/H782Q variant. On the other hand, <10% of the interactions for *L*. *lactis* A and the E652D/H782Q/K786N variant present forces greater than 900 pN when interacting with Fg.

For the experiments shown in Fig. 6b, Fg was linked to the tip through non-specific adsorption similar to what likely occurs *in vivo*. We also performed experiments by using NHS chemistry to specifically link Fg to the AFM tip. A similar bimodal binding force distribution (i.e., a group of weak forces of ~250 pN in 29% of interactions and group of forces centered at ~1.3 nN in 71% of the interactions) was observed for the E652D/H782Q:Fg interaction (Fig. S5a). This demonstrates that the measured forces were not dependent on the ligand grafting protocol, and confirms data shown in Fig. 6b.

When comparing the static microtiter to dynamic AFM assay, there is an apparent strengthening of adhesion observed for the E652D/H782Q variant with Fg in most of the interactions. For this variant, a weaker interaction relative to the wild type (*L*. *lactis* A) was observed in the microtiter assay (Fig. 3b) whereas a remarkably stronger bond compared to the wild type was measured in the AFM (Fig. 6a). This means that induced force affects FnBPA-Fg interactions for the E652D/H782Q variant bond. These findings suggest a different binding mechanism than that observed for Fn.

To test if the strength of the FnBPA-Fg bond varies with the rate at which force is applied, *i*.*e*., loading rate, we plotted these parameters in Fig. 6c. This figure shows singular distributions from the different pulling velocities (different data colors in Fig. 6c). This unbinding distribution for Fg is quite different than the single distribution observed for Fn (Fig. S6). Similar distributions of force obtained at different pulling velocities were observed with Fg attached to the AFM tip through NHS chemistry (Fig. S5b) indicating neither the tip surface chemistry nor ligand grafting protocol impacted the observed force measurements. Furthermore, these results confirm a different response to tensile loading for these two host ligands (Fn vs. Fg).

**Figure 7:**
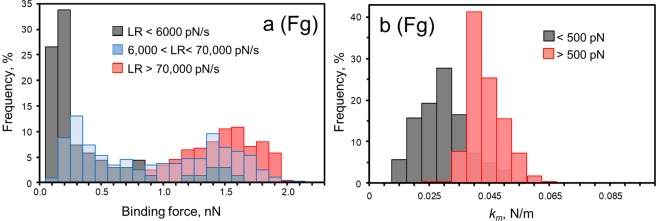
(**a**) Force distributions of the Fg-FnBPA interactions over different ranges of loading rates (LR). The probability of forming strong bonds increases with the rate of the load. LR values were binned <6,000 pN/s (gray bars), from 6,000 to 70,000 pN/s (blue bars), and >70,000 pN/s (red). (**b**) Distribution of the spring constant of the molecular Fg-FnBPA complex (*k*_*m*_) at low (<500 pN, gray bars) and high (>500 pN, red bars) forces. For these experiments, AFM tips were coated with protein by incubating tips in 100 µg/mL of Fn or Fg prior to force measurements.

Because of the multiple unbinding distributions, it is a challenge to model the Fg-FnBPA bond using the Bell model as was performed for Fn. Therefore, an alternative analysis of the Fg-FnBPA bond was conducted by examining the distributions over different ranges of loading values (Fig. 7a). We observed a significant switch in the force distribution with the loading rate. At a slow rate, the majority (>60%) of the forces were weak (≤200 pN), whereas at a fast load, most of the forces (>69%) were strong (≥1200 pN). Both weak and strong forces were seen at intermediate loading rates. Comparable distributions of force at different loading were observed for Fg attached to the AFM tip through NHS chemistry (Fig. S5c). The transition from weak to strong binding correlated with an increase in molecular stiffness (*k*_*m*_) of the Fg-FnBPA bond (Fig. 7b)^22,30^. Weak and strong forces presented average values of *k*_*m*_ = 27 pN/nm and 41 pN/nm, respectively. These findings were only observed for the Fg-FnBPA variant with the E652D/H782Q substitutions.

## Discussion

Fibronectin- and fibrinogen-binding MSCRAMMs in *S*. *aureus* promote bacterial adhesion to host tissues targeting Fn^31,32^ or Fg^9^. These proteins also promote attachment to inert surfaces conditioned with host plasma proteins^33^. *S*. *aureus* adhesion to cells, tissues and devices is a critical first step in the infection process^9,18,34^. Binding of FnBPA to Fg and Fn has been shown to be associated with infection in clinical studies and animal models^9,35,36^.

In this study, we evaluate ligand interactions with FnBPA variants that have amino acid substitutions in the FnBRs (Fig. 1a) previously shown to be associated with infections of cardiovascular devices^14,15,17^. These substitutions in FnBPA were expressed in *L*. *lactis*, to avoid other adhesins present in *S*. *aureus* that could complicate the interpretation of the results. The FnBPA variants were tested for their ability to bind to Fn and Fg using both a microtiter assay, a commonly used static, indirect measure of bacterial adhesion, and AFM, a technique that dynamically measures the actual force of bond strength. The impact of mechanical stress on adhesion was accomplished by the comparison of the results from these two techniques.

For Fn, single and multiple substitutions enhanced binding in the majority (5 of 7) of the FnBPA variants tested in the microtiter assay (Fig. 3a). The E652D/H782Q and E652D/H782Q/K786N variants tested with AFM also showed greater bond strength (Fig. 5a). These results with *L*. *lactis* are consistent with previous reports for isolates of *S*. *aureus* from patients with cardiac infections^14–16^ and with peptides that resemble FnBRs in FnBPA^37^. From these previous studies, it was concluded that amino acid substitutions in the FnBR region of FnBPA lead to increased bond strength with Fn and influence the conformation of Fn upon binding.

Dissociation rate constants (*k*_*off*_) for Fn-FnBPA interactions could be determined because this ligand-receptor pair followed dissociation pathways described by the Bell-Evans model (Figs 5b and S6). The dissociation rate constants (*k*_*off*_) values determined by this model range from 1.4 to 4.0 s^−1^ and are comparable to dissociation rate constants determined for bacterial adhesion to other human proteins, including; *Burkholderia cepacia* and collagen, *k*_*off*_ = 2.8 s^−1^ ^38^; and *S*. *epidermidis* and Fn, *k*_*off*_ = 4.76 s^−1^ ^39^. The *k*_*off*_ values obtained here for full-length FnBPA are also comparable to off-rates determined for 20-mer peptides carrying amino acid substitutions designed to mimic FnBR-9, a repeat linked to infections of cardiac devices (*k*_*off*_ = 5.2 to 5.9 s^−1^)^37^.

Nonlinear trends in rupture force with loading rate are typically explained in conventional models by invoking two (or more) barriers along the unbinding pathway^25^. Multiple barriers, defined by the position(s) of the activation energy barrier (*x*_*β*_), often emerge along the energy landscape^25,39^. Barriers characterized by large *x*_*β*_ are considered to be more elastic and able to sustain low forces over larger deformations^39^. Conversely, short *x*_*β*_ barriers can sustain high stress for short loading times^39^. We have taken this approach here (see Fig. 5b) to facilitate comparison to past studies that have also used AFM to mechanically induce dissociation with *S*. *aureus* MSCRAMM proteins^17,21,37,40–42^. It is worth mentioning that a recent alternative model describes nonlinear trends in force with loading rate as two regimes of bond rupture (a near-equilibrium regime and kinetic regime)^43–45^.

For our studies with Fn, a longer bond survival time (average 1/*k*_*off*_ = 0.5 s) was observed for the outer barrier (*x*_*β*_ > 0.2 Å, lower-strength regime Fig. 5b). Whereas, a shorter bond lifetime (average 1/*k*_*off*_ = 0.37 s) was measured for the inner barrier (*x*_*β*_ < 0.2 Å, higher-strength regime Fig. 5b). A similar relationship between inner/outer barriers and short/long bond lifetimes was observed for interactions between Fn and an Fn-adhesin in *S*. *epidermidis*^39^. For the data shown herein, interactions of Fn with wild-type FnBPA exhibited the shortest lifetime compared to that for the E652D/H782Q variant in the lower-strength regime, and to that for the E652D/H782Q/K786N variant in the higher-strength regime.

Overall, the *k*_*off*_ values for the E652D/H782Q and E652D/H782Q/K786N variants indicate a stronger interaction with Fn compared to the wild-type FnBPA. For example, the bond lifetime (1/*k*_*off*_) for E652D/H782Q/K786N is ~2x longer compared to the wild-type. This increase in bond lifetime is remarkably similar to prior Fn-binding studies with *S*. *aureus* expressing FnBPA with E652D/H782Q/K786N substitutions. The enhancement was 1.86x stronger (*k*_*off*_ = 0.56 sec^−1^) compared to strains mostly lacking the substitutions (*k*_*off*_ = 1.04 sec^−1^)^17^.

Switching to the other ligand, the microtiter adhesion assay demonstrated that binding to Fg was impacted by amino acid substitutions in the repeat region of FnBPA. Binding to Fg was diminished in all seven FnBPA variants tested with the microtiter assay (Fig. 3b). AFM experiments also demonstrated that Fg bond strength was affected by the presence of these substitutions (Fig. 6). These results were unexpected for the Fg ligand since the FnBR-5 and -9 substitutions are, for the primary linear configuration, distant from the known Fg-binding site in the A domain of FnBPA. The FnBPA-Fg interaction involves the N2N3 subdomains (see Fig. 1a) through a variant of the DLL mechanism^46,47^. The microtiter and AFM results shown herein suggest that the unstructured FnBRs interact with Fg, possibly folding back on the A-domain in a way that impacts the DLL mechanism.

Perhaps the most important observation for Fg was the dramatic >5-fold increase in bond strength for the E652D/H782Q variant (Fig. 6a), which suggests a different binding mechanism. This single FnBPA-Fg bond is remarkably strong (~1.3 nN), similar to the strength of a covalent bond^48^. A similarly strong binding force of ~2 nN was reported for Fg binding to SdrG (a *S*. *epidermidis* cell-wall protein). This Fg-SdrG bond followed a DLL binding mechanism involving a conformational change of the adhesin resulting in a greatly stabilized complex^30,49^. Contrary to this, a different, non-DLL mechanism between Fg and the integrin α_IIb_β_3_ have shown weaker binding forces of 20–150 pN^50,51^.

The AFM results indicate, particularly for the E652D/H782Q substitution, different binding mechanisms for FnBPA interacting with Fg. There is a group of weak forces at ~200 pN, and a second group that exhibits stronger binding forces (~1.3 nN) (Figs 6b and 7a). We propose that the strong forces arise from interactions where a conformation change in binding from a weak (~200 pN) to a strong binding state has occurred. This transition from weak to strong forces correlates with an increase in molecular stiffness of the protein complex (Fig. 7b). It seems that under mechanical tension and extension of the FnBPA molecule, there is a conformation change that favors a stronger interaction with Fg. This molecular modification is not triggered for the static microtiter assays.

Bacteria certainly experience different physical stresses within the AFM vs. the microtiter environment. In this study, we are interested in the stress that bacteria may experience in flowing solution within the circulatory system. Estimates suggest that bacterial cells experience loads in excess of 10^4^ to 10^6^ pN/s in flowing blood^21^. Our results demonstrate that loading rates within this range (Figs 7a and S5c) trigger a strong binding reaction. This suggests that force-induced adhesion to Fg will occur for FnBPA expressed on the cell wall of *S*. *aureus* cells *in vivo*. Furthermore, these findings reveal the importance of performing binding studies under mechanical stress to better mimic bacterial adhesion under shear that is likely *in vivo*. This force-activated behavior for Fg also means that care must be taken when interpreting Fg binding results from different adhesion assays. The binding conditions (static vs. dynamic) of the assay can lead to different conclusions when Fg is the ligand.

This observed strengthening of the Fg-bond in the presence of a tensile force (Fig. 6a) is reminiscent of catch-bond behavior. A catch-bond is a bond that is enhanced by mechanical force^52^ such that the dissociation lifetime of this noncovalent bond increases with the applied force. In this counterintuitive behavior, the external force prolongs bond lifetime. The FimH-mannose bond in *Escherichia coli* is a well-documented example of a catch bond. At a low flow, this bond is weak and relatively short lived, whereas at high flow the bond is stronger. The allosteric model proposes that force triggers an allosteric switch from low- to high-affinity conformation of the adhesin^53^. Specifically, it has been reported that catch bonds arise due to force-induced remodeling of the hydrogen bond networks^54,55^. Perhaps the FnBPA-Fg interaction shown here involves an allosteric conformational change from low to high-affinity state when force is applied. Although strong forces were detected in all the different FnBPA variants including the wild-type, the fact that most of the interactions of Fg and E652D/H782Q variant were in the nanonewton range suggests that the two substitutions E652D and H782Q may alter hydrogen bond networks favoring a higher affinity state. Indeed, others have shown that even a, single point mutation can dramatically alter the nature of the catch bond compared to the wild type^54^.

Alternative explanations for force-enhanced adhesion besides catch bonds have been proposed for other bacteria. In *Pseudomonas aeruginosa*, for example, properties of the primary surface appendages (pili, flagellum, and exopolysaccharide) may not regulate the shear-enhanced adhesion. Instead, enhanced attachment of *P*. *aeruginosa* in shear stress is thought to originate from intrinsic features of the cell surface and/or a dynamic cell response^56^. Other studies in *Bacillus subtilis* and *P*. *aeruginosa*, support this idea that flow encourages sessile over free-swimming styles due to sensing of chemical signals by planktonic microbes, which is inhibited by flow. In these cases, attachment is thought to be enhanced independent of specific adhesion properties^57^.

Forces in the nanonewton range may arise from multiple interactions that could explain the multiple populations seen in Fig. 6c. However, the narrow force distribution at ~1.3 nN (Fig. 6b) suggests that the strong forces (~1.3 nN) arise from single FnBPA-Fg interactions. When multiple bonds break simultaneously, intermediate forces arising from multiples of the weakest force unit are typically observed, resulting in more complex force distributions^21^. A similar, narrow force distribution was also observed using NHS chemistry to attach Fg molecular onto the AFM tip (Fig. S5a). This attachment protocol is known to favor single-molecule interactions^21^. Furthermore, binding studies with ClfA (a *S*. *aureus* protein with a similar Fg binding A-region) have reported a similar force-enhanced adhesion towards Fg, suggesting that this ligand exhibits this behavior with multiple proteins^21^.

Additionally, a growing number of binding studies with staphylococcal adhesins have shown that these proteins interact with their selected target protein with resilient mechanostability withstanding forces of ~1.5 to ~2 nN^22,23,30,49,58^. The origin of this stability has been recently described for the SdrG-Fg system and it comes from an intricate hydrogen bond network between the ligand and the receptor. In this interaction, the DLL mechanism creates a binding pocket confining the target in a stable geometry. It has been suggested that this binding mechanism may be generalized to other *S*. *aureus* adhesins including FnBPA and Fg^49^.

Collectively, the Fn- and Fg-binding experiments in our study help shed light on the fundamental causes of *S*. *aureus* infections in humans. Six to nine Fn molecules can bind to a single FnBPA molecule^12^. This multivalent capability of FnBPA suggests that Fn should dictate adhesion of *S*. *aureus* to biomaterials in contact with blood, unless binding is sterically hindered by a Fg molecule bound to the A-domain of FnBPA^47^. This inhibition is possible as the plasma concentration of Fg is 10-fold higher than Fn, and thus more Fg may be on indwelling materials. Predominance of Fg has been reported for biomaterials in contact with blood for short periods of time (~2 weeks). Yet Fn is often reported to be more active than Fg in promoting *S*. *aureus* adherence to biomaterials^8^, in part due to the inactivation/proteolysis of Fg when it adsorbs onto artificial surfaces^59^. Therefore, it may be that Fg is the more active component for initial adhesion *in vivo*, whereas Fn is more active for long term implants^36,60^, as in the case of cardiac devices. Still, Fg-mediated adhesion should not be ignored particularly under shear conditions due to its ability to form force-enhanced adhesion, and therefore this protein complex is capable of withstanding forces equivalent to the strength of a covalent bond.

Another aspect to consider is the rate of evolution of virulence factors for clinical infections. FnBPA has two distinct binding segments, a structured A-domain and disordered repeat region (Fig. 1a), which may evolve at different rates. Disordered regions are highly flexible and therefore expected to “respond” more quickly than structured regions in a protein^61^. Amino acid substitutions in the disordered FnBRs could evolve in response to changes in the physiological surroundings of *S*. *aureus*, which include Fn- and/or Fg associated with indwelling devices or blood clots. This may partly explain the enhancement in adhesion observed for both ligands in the E652D/H782Q variant under dynamic conditions. Additionally, these substitutions could evolve to self-regulate the adherence for one ligand over another as seen in the E652D/H782Q/K786N variant, which showed enhanced adherence for Fn but diminished adherence for Fg under dynamic conditions. This self-regulatory mechanism is similar to the negative binding regulation that occurs when a single FnBPA molecule simultaneously binds to both Fn and Fg^47^.

In summary, variants of full-length FnBPA have been expressed in the proper conformation and orientation on *L*. *lactis*. Experiments have shown that FnBPA exhibits ligand-dependent adhesive behavior. In the case of Fn, substitutions E652D, H782Q, and K786N in FnBR-5 and FnBR-9 result in stronger more resilient bonds under both static and dynamic conditions. The binding landscape for Fn-FnBPA follows a single dissociation pathway that is well described by the Bell-Evan model. Conversely, Fg binding to FnBPA depended on surrounding conditions (static vs. dynamic). When subjected to a tensile force, the FnBPA-Fg interaction was remarkably strong, particularly for the E652D/H782Q variant. This indicates that Fg molecules (more than Fn) are susceptible to conformation changes that impact binding. When external mechanical stress was applied to the Fg-E652D/H782Q interaction, there was a force-enhanced adhesion. Observed changes in binding to Fg were unexpected because the Fg-binding site in FnBPA (i.e., the A-domain) is distant from the position of the substitutions, at least for the linear configuration of FnBPA. This study reveals that the activation of FnBPA-Fg interaction during mechanical stress could enable *S*. *aureus* to withstand high shear flow during colonization.

## Methods

### Bacterial strains and growth conditions

Cryopreserved *L*. *lactis* strains were grown to exponential phase (OD_550nm_ = 0.54 ± 0.04) at 30 °C in M17 supplemented with erythromycin and 0.5% dextrose, harvested and then washed in PBS. The cell concentration was adjusted to an OD_600nm_ = 1.0 corresponding to 10^7^ cells/ml for the 96-wells plate adhesion and optical microscopic images. The AFM measurements and the optical microscopic images were acquired within 2 hours after harvesting the cells to ensure cell viability^62^.

### Static adhesion assay

The microtiter assay was slightly modified from Lower *et al*.^14^. Briefly, 96-well plates were coated with Fn or Fg in PBS (pH 7.4). The plates were washed with PBS, blocked with 2% BSA solution, and washed again. Washed cells were incubated in the wells, washed in PBS, fixed with 2.5% formaldehyde, followed by 0.5% crystal violet, and DMSO. Absorbance was measured at 620 nm using with a plate reader (BioTek). These experiments were conducted at least in triplicate for all the bacteria strains.

For light microscopy, washed cells were deposited on protein-coated glass for 10 min, gently rinsed with PBS then observed in to an optical microscope (Axiovert 200M, Zeiss). Optical images were collected on at least three spots on a glass slide from three independent cell cultures.

### Atomic force spectroscopy

A Bioscope Resolve AFM (Bruker) was used for force measurements. An attached inverted microscope (Axiovert 200M; Zeiss) was used to position the AFM tip over *L*. *lactis* cells. A total of 352 different *L*. *lactis* from 57 independent cell cultures were probed. Si_3_N_4_ probes with nominal tip radius 20 nm, and spring constant 0.076 nN nm^−1^ (thermal tuning method) were coated with Fn or Fg according to published protocol^14,17^. Briefly, a clean AFM tip was coated with Fn and Fg by immersion in a 100 µg/ml Fn or Fg PBS solution for 45 min, and then rinsed in PBS. Both Fn and Fg were deposited through this non-specific method, to mimic the conditions in the human body where these blood proteins coat the surface of cardiac implants through non-specific interactions. Fg molecules were also immobilized on the cantilever at low density using N-hydroxysuccinimide (NHS) surface chemistry following reported protocols^21^ to favor single molecule detection^63^.

A total of 55 different tips were used. AFM measurements were conducted in PBS, at retraction velocities from 0.05 to 18.8 µm/s for both Fn and Fg experiments generating over 200,000 force curves. To ensure similar concentration of binding pairs for the various *L*. *lactis* strains, force spectra with comparable binding frequency were included in the post-collection analysis. This approach also helped ensure Fn/Fg were not denatured or inactivated, something that has been reported for low concentration of the ligand on the AFM tip^27^. For nonspecific binding of ligands to the tip, median binding frequencies of the all the protein complexes analyzed was <46% for Fn and <60% for Fg. In the case of studies with Fg immobilized on the cantilever through specific methods (NHS chemistry), the median binding frequency was 49%. To ensure specificity, only the final binding peak was included in the analyses in all the studies. Control experiments were conducted with an uncoated AFM tip. Additionally, control studies for specific interactions between Fn-FnBPA and Fg-FnBPA were monitored from successive binding events with a peak-to-peak distance (ΔL) from the unfolding distance of multiple repeats in Fn (30 ± 4 nm) and structural elements in Fg (36 ± 10 nm). Specifically, the ΔL values were revised from force spectra collected at the beginning and the end of each experiment. That is, at the start and after several hundred interactions on each *L*. *lactis* cell probed in each experiment. Each AFM tip was used on only a few cells before being discarded when they no longer exhibited these unfolding patterns (ΔL values). Control studies conducted with NHS-modified cantilevers presented similar ΔL for the Fg-FnBPA interaction. Negative controls were performed using *L*. *lactis* cells with an empty plasmid (*L*. *lactis* 0).

## Supplementary information

Supplemental Information

## References

[1] Foster TJ, Geoghegan JA, Ganesh VK, Hook M (2014). Adhesion, invasion and evasion: the many functions of the surface proteins of *Staphylococcus aureus*. Nat. Rev. Microbiol..

[2] Herrmann M (1988). Fibronectin, fibrinogen, and laminin act as mediators of adherence of clinical staphylococcal isolates to foreign material. J. Infec.Dis.

[3] Vaudaux P (1989). Host factors selectively increase staphylococcal adherence on inserted catheters - a role for fibronectin and fibrinogen or fibrin. J. Infec. Dis..

[4] Ariens RAS (2013). Fibrin(ogen) and thrombotic disease. J. Thromb. Haemost..

[5] Lowe GDO, Rumley A, Mackie IJ (2004). Plasma fibrinogen. Ann. Clin. Biochem..

[6] Mosher DF (2006). Plasma fibronectin concentration - A risk factor for arterial thrombosis?. Arterioscler. Thromb. Vasc. Biol..

[7] Mosher DF (1984). Physiology of fibronectin. Annu. Rev. Med..

[8] Vaudaux P (1993). Fibronectin is more active than fibrin or fibrinogen in promoting *Staphylococcus-aureus* adherence to inserted intravascular catheters. J. Infec. Dis..

[9] Que YA (2005). Fibrinogen and fibronectin binding cooperate for valve infection and invasion in *Staphylococcus aureus* experimental endocarditis. J. Exp Med..

[10] Ganesh VK (2008). A structural model of the *Staphylococcus aureus* ClfA-fibrinogen interaction opens new avenues for the design of anti-staphylococcal therapeutics. PLoS Pathog..

[11] Ponnuraj K (2003). A “dock, lock, and latch” structural model for a staphylococcal adhesin binding to fibrinogen. Cell.

[12] Bingham RJ (2008). Crystal structures of fibronectin-binding sites from *Staphylococcus aureus* FnBPA in complex with fibronectin domains. Proc. Natl. Acad. Sci. USA.

[13] Schwarz-Linek U (2003). Pathogenic bacteria attach to human fibronectin through a tandem beta-zipper. Nature.

[14] Lower SK (2011). Polymorphisms in fibronectin binding protein A of *Staphylococcus aureus* are associated with infection of cardiovascular devices. Proc. Natl. Acad. Sci. USA.

[15] Hos NJ (2015). Amino acid alterations in fibronectin binding protein A (FnBPA) and bacterial genotype are associated with cardiac device related infection in *Staphylococcus aureus* bacteraemia. J. Infect..

[16] Xiong YQ (2015). Endovascular infections caused by methicillin-resistant *Staphylococcus aureus* are linked to clonal complex-specific alterations in binding and invasion domains of fibronectin-binding protein a as well as the occurrence of fnbB. Infect. Immun..

[17] Casillas-Ituarte NN, Lower BH, Fowler VG, Lamlertthon S, Lower SK (2012). Dissociation rate constants of human fibronectin binding to fibronectin-binding proteins on living *Staphylococcus aureus* isolated from clinical patients. J. Biol. Chem..

[18] Que YA (2001). Reassessing the role of *Staphylococcus aureus* clumping factor and fibronectin-binding protein by expression in *Lactococcus lactis*. Infect. Immun..

[19] Que YA, Haefliger JA, Francioli P, Moreillon P (2000). Expression of *Staphylococcus aureus* clumping factor A in *Lactococcus lactis* subsp *cremoris* using a new shuttle vector. Infect. Immun..

[20] Persat A (2015). The mechanical world of bacteria. Cell.

[21] Herman-Bausier P (2018). *Staphylococcus aureus* clumping factor A is a force-sensitive molecular switch that activates bacterial adhesion. Proc. Natl. Acad. Sci. USA.

[22] Vitry P (2017). Force-induced strengthening of the interaction between *Staphylococcus aureus* clumping factor B and Loricrin. mBio.

[23] Geoghegan JA, Dufrene YF (2018). Mechanomicrobiology: how mechanical forces activate *Staphylococcus aureus* adhesion. Trends Microbiol..

[24] Bell G (1978). Models for specific adhesion of cells to cells. Science.

[25] Evans E (2001). Probing the relation between force - Lifetime - and chemistry in single molecular bonds. Ann. Rev. Bioph. Biom.

[26] Schneewind O, Mihaylovapetkov D, Model P (1993). Cell-wall sorting signals in surface-proteins of gram-positive bacteria. Embo J..

[27] Meadows PY, Bemis JE, Walker GC (2003). Single-molecule force spectroscopy of isolated and aggregated fibronectin proteins on negatively charged surfaces in aqueous liquids. Langmuir.

[28] Oberhauser AF, Badilla-Fernandez C, Carrion-Vazquez M, Fernandez JM (2002). The mechanical hierarchies of fibronectin observed with single-molecule AFM. J. Mol. Biol..

[29] Zhmurov A (2011). Mechanism of fibrin(ogen) forced unfolding. Structure.

[30] Herman P (2014). The binding force of the staphylococcal adhesin SdrG is remarkably strong. Mol. Microbiol..

[31] Hauck CR, Ohlsen K (2006). Sticky connections: extracellular matrix protein recognition and integrin-mediated cellular invasion by *Staphylococcus aureus*. Curr. Opin. Microbiol..

[32] Sinha B (1999). Fibronectin-binding protein acts as *Staphylococcus aureus* invasin via fibronectin bridging to integrin alpha(5)beta(1). Cell Microbiol..

[33] Vroman L, Adams AL (1986). Adsorption of proteins out of plasma and solutions in narrow spaces. J. Colloid Interface Sci..

[34] Shinji H (2011). Role of Fibronectin-binding proteins A and B in *in vitro* cellular infections and *in vivo* septic infections by *Staphylococcus aureus*. Infect. Immun..

[35] Kuypers JM, Proctor RA (1989). Reduced adherence to traumatized rat-heart valves by a low-fibronectin-binding mutant of *Staphylococcus aureus*. Infect. Immun..

[36] Piroth L (2008). The fibrinogen- and fibronectin-binding domains of *Staphylococcus aureus* fibronectin-binding protein a synergistically promote endothelial invasion and experimental endocarditis. Infect. Immun..

[37] Casillas-Ituarte NN (2017). Amino acid polymorphisms in the fibronectin-binding repeats of fibronectin-binding protein A affect bond strength and fibronectin conformation. J. Biol. Chem..

[38] El-Kirat-Chatel S, Mil-Homens D, Beaussart A, Fialho AM, Dufrene YF (2013). Single-molecule atomic force microscopy unravels the binding mechanism of a *Burkholderia cenocepacia* trimeric autotransporter adhesin. Mol. Microbiol..

[39] Bustanji Y (2003). Dynamics of the interaction between a fibronectin molecule and a living bacterium under mechanical force. Proc. Natl. Acad. Sci. USA.

[40] Herman-Bausier P, El-Kirat-Chatel S, Foster TJ, Geoghegan JA, Dufrene YF (2015). *Staphylococcus aureus* fibronectin-binding protein a mediates cell-cell adhesion through low-affinity homophilic bonds. mBio.

[41] Herman-Bausier, P. *et al*. Mechanical strength and inhibition of the *Staphylococcus aureus* collagen-binding protein Cna. *mBio* **7**, 10.1128/mBio.01529-16 (2016). 10.1128/mBio.01529-16 PMC5080380 27795393

[42] Mitchell G (2008). *Staphylococcus aureus* SigB activity promotes a strong fibronectin-bacterium interaction which may sustain host tissue colonization by small-colony variants isolated from cystic fibrosis patients. Mol. Microbiol.

[43] Friddle RW, Noy A, De Yoreo JJ (2012). Interpreting the widespread nonlinear force spectra of intermolecular bonds. Proc. Natl. Acad. Sci. USA.

[44] Sand KK, Friddle RW, DeYoreo JJ (2017). Quantifying the free energy landscape between polymers and minerals. Sci. Rep.

[45] Alsteens D (2015). Imaging G protein-coupled receptors while quantifying their ligand-binding free-energy landscape. Nat. Methods.

[46] Edwards AM, Potts JR, Josefsson E, Massey RC (2010). *Staphylococcus aureus* host cell invasion and virulence in sepsis is facilitated by the multiple repeats within FnBPA. PLoS Pathog..

[47] Stemberk V (2014). Evidence for steric regulation of fibrinogen binding to *Staphylococcus aureus* fibronectin-binding protein A (FnBPA). J. Biol. Chem..

[48] Grandbois M, Beyer M, Rief M, Clausen-Schaumann H, Gaub HE (1999). How strong is a covalent bond?. Science.

[49] Milles LF, Schulten K, Gaub HE, Bernardi RC (2018). Molecular mechanism of extreme mechanostability in a pathogen adhesin. Science.

[50] Litvinov RI, Bennett JS, Weisel JW, Shuman H (2005). Multi-step fibrinogen binding to the integrin alpha llb beta 3 detected using force spectroscopy. Biophys. J..

[51] Agnihotri A, Soman P, Siedlecki CA (2009). AFM measurements of interactions between the platelet integrin receptor GPIIbIIIa and fibrinogen. Colloid Surf. BBiointerfaces.

[52] Sokurenko EV, Vogel V, Thomas WE (2008). Catch-bond mechanism of force-enhanced adhesion: counterintuitive, elusive, but… widespread?. Cell Host Microbe.

[53] Yakovenko O (2008). FimH forms catch bonds that are enhanced by mechanical force due to allosteric regulation. J. Biol. Chem..

[54] Chakrabarti S, Hinczewski M, Thirumalai D (2014). Plasticity of hydrogen bond networks regulates mechanochemistry of cell adhesion complexes. Proc. Natl. Acad. Sci. USA.

[55] Kong F, Garcia AJ, Mould AP, Humphries MJ, Zhu C (2009). Demonstration of catch bonds between an integrin and its ligand. J. Cell Biol..

[56] Lecuyer S (2011). Shear stress increases the residence time of adhesion of *Pseudomonas aeruginosa*. Biophys. J..

[57] Rusconi R, Guasto JS, Stocker R (2014). Bacterial transport suppressed by fluid shear. Nature Physics.

[58] Herman-Bausier P, Dufrene YF (2018). Force matters in hospital-acquired infections extremely strong forces help staphylococci to colonize biomaterials and infect humans. Science.

[59] Brash JL, Thibodeau JA (1986). Identification of proteins absorbed from human-plasma to glass bead columns - plasmin-induced degradation of adsorbed fibrinogen. J. Biomed. Mater. Res..

[60] Francois P, Vaudaux P, Foster TJ, Lew DP (1996). Host-bacteria interactions in foreign body infections. Infect. Control Hosp. Epidemiol..

[61] Dunker AK, Obradovic Z (2001). The protein trinity - linking function and disorder. Nat. Biotechnol..

[62] Boonaert CJP, Dufrene YF, Derclaye SR, Rouxhet PG (2001). Adhesion of *Lactococcus lactis* to model substrata: direct study of the interface. Colloid Surf. B-Biointerfaces.

[63] Berquand A (2005). Antigen binding forces of single antilysozyme Fv fragments explored by atomic force microscopy. Langmuir.

