# Supplementary material for: Fibrinogen binding is affected by amino acid substitutions in C-terminal repeat region of fibronectin binding protein A
Source: Sci Rep. 2019 Aug 12;9:11619. doi: 10.1038/s41598-019-48031-5 (PMC6690874; doi:10.1038/s41598-019-48031-5)
Supplement: Supplementary file 1 — Supplemental Information [file 41598_2019_48031_MOESM1_ESM.pdf]

Supplementary Information

Fibrinogen binding is affected by amino acid substitutions in C-terminal repeat region of fibronectin binding protein A

Nadia N. Casillas-Ituarte, Alex C. DiBartola, Megan J. Broughton, Lumarie Pérez-Guzmán, Robert M. Wheeler, Makoto Ibaraki, B. Alexis Lower, James A. Dunn, Brian H. Lower, Vance G. Fowler Jr, Magnus Höök, Lauren M. McIntyre, Steven K. Lower, Batu K. Sharma-Kuinkel

Supplementary Methods

Growth curves

Cryopreserved *L. lactis* strains were grown at 30° C in M17 supplemented with erythromycin and 0.5% dextrose. Absorbance readings at 600 nm were collected approximately every 50 min for >10 hrs.

FnBPA expression on *L. lactis*

The expression of *S. aureus* adhesins, including FnBPA, in *L. lactis* has been described previously [1,2]. Briefly, *Lactococcus lactis* subsp. *cremoris 1363* was used to express full-length FnBPA. This expression was accomplished with an *Escherichia coli-L. lactis* shuttle and expression vector. The lactococcal plasmid pIL253 was equipped with the *oriColE*1 replicon to generate pOri253 that could replicate in *E. coli*. Then, the promoter P23 was inserted in pOri253 to generate pOri23. The *fnbA* gene was cloned into pOri23 to produce pOri23-*fnbA* which was inserted in *L. lactis.*

Flow cytometry

Surface expression of FnBPA in *L. lactis* was measured by using flow cytometry as described previously[3]. Briefly, *Lactococcus lactis* strains expressing each of seven full-length FnBPA variants with amino acid substitutions in FnBR-5 (E652D) and/or FnBR-9 (H782Q, K786N) were grown to mid log phase, washed 1X with PBS and blocked with R347 blocking reagent for 60 min at 4 ˚C. The cells were then incubated with primary antibody (α-FnbpA – polyclonal rabbit sera) for 30 min at 4 ˚C followed by washing with FACS wash buffer (0.1% Tween 20/0.1% BSA in 1XPBS). Finally, the

cells were stained with Goat Anti-Rabbit Alexa 647 secondary antibody (Invitrogen, Carlsbad, CA, USA), incubated for 30 minutes at 4 °C, washed several times with FACS buffer, re-suspended in FACS buffer. Since this study involves just a single population, the gating was set to include a desired number of cells to analyze. The diagrams (SI Figure S3) show two peaks, the one of the left side is assigned to unstained controls (no FnBPA expressing cells), whereas the peak on the right side arises from the cell population expressing FnBPA. Unstained control was used to adjust the voltage, so the inflection point on the right side of the curve is around 10^2. A total of 30,000 events were collected per sample. Data were acquired using a BD FACS Canto IIs analyzer at Duke Flow Cytometry Core. Acquired data were analyzed with FlowJo.

Western ligand blots

Surface expression of FnBPA in *L lactis* was determined by ligand affinity blotting by incubation with pure human Fn as described by Que *et al.,* [2] and Bisognano *et al.* [4]. Briefly, cell wall associated proteins were extracted from 500 ml portions of mid-log phase cultures washed twice in ice-cold 0.9 M NaCl, and resuspended in 5 ml of digestion buffer (1.1 M sucrose, 1mM $CaCl_2$, 0.5 mM in $MgCl_2$ in PBS) in the presence of 100 µg/ml of RNase, 50 µg/ml of DNase, a cocktail of antiproteases (Pierce), 5 mg/ml of lysozyme (Sigma), and 100 µg/ml of mutanolysin (Sigma). Protein was determined using the BCA method. The equivalent of 10 µg of protein per sample was separated by SDS-PAGE (12% separating gel) and transferred overnight (at 4 °C) onto polyvinylidene difluoride membranes (Invitrogen).

Membranes were blocked with StartingBlock T20 (TBS) blocking buffer (Invitrogen). Blots were incubated with 30 μg/ml Fn (Sigma) for 1 h followed by an anti-N-terminal Fn antibody (1:3,000; Novus, Biologicals) for 1 h. The secondary antibody was anti-mouse IgG coupled to peroxidase (R&D System). Detection was performed with an ECL kit (Pierce). FnBPA was identified in the reference *L. lactis A* cells. *L. lactis 0* cells were used as a negative control. Total FnBPA concentrations were estimated with densitometry by ImageJ (version 1.49v). Band density values were normalized to the band expressed on the reference wild type *L. lactis A.*

Estimate number of Fg and Fn molecules on slides and microtiter plates

The glass slides were coated with 40 μl of 100 μg/ml solution of Fg or Fn. Using the molecular weight for each ligand, the estimated number of molecules on the glass slides were 7.30×10^{12} and 5.48×10^{12} for Fg and Fn, respectively.

References

1 Que, Y. A. *et al*. Reassessing the role of *Staphylococcus aureus* clumping factor and fibronectin-binding protein by expression in *Lactococcus lactis*. *Infect. Immun.* **69**, 6296-6302 (2001).

2 Que, Y. A., Haefliger, J. A., Francioli, P. & Moreillon, P. Expression of *Staphylococcus aureus* clumping factor A in *Lactococcus lactis* subsp *cremoris* using a new shuttle vector. *Infect. Immun.* **68**, 3516-3522 (2000).

3 Tkaczyk, C. *et al.* Targeting Alpha Toxin and ClfA with a Multimechanistic Monoclonal Antibody-Based Approach for Prophylaxis of Serious Staphylococcus aureus Disease. *mBio* **7**, 11, doi:10.1128/mBio.00528-16 (2016).

4 Bisognano, C., Vaudaux, P., Rohner, P., Lew, D. P. & Hooper, D. C. Induction of fibronectin-binding proteins and increased adhesion of quinolone-resistant Staphylococcus aureus by subinhibitory levels of ciprofloxacin. *Antimicrobial Agents and Chemotherapy* **44**, 1428-1437 (2000).

Supplementary Table S1. Median force binding values for *L. lactis* strains and both ligands (Fn, top; Fg, bottom), as determined by AFM. *L. lactis* 0 is the negative control lacking FnBPA on the cell wall. P-values, relative to *L. lactis* A, calculated using Mann-Whitney test.

**Fn**	***L. lactis A***	**E652D+H782Q**	**E652D+H782Q+K786N**	***L. lactis 0***
**median**	0.216	0.238	0.227	0.117
***p* values**	-	0.765	0.281	<0.0001

**Fg**	***L. lactis A***	**E652D+H782Q**	**E652D+H782Q+K786N**	***L. lactis 0***
**median**	0.241	1.339	0.209	0.162
***p* values**	-	<0.0001	0.188	<0.0001

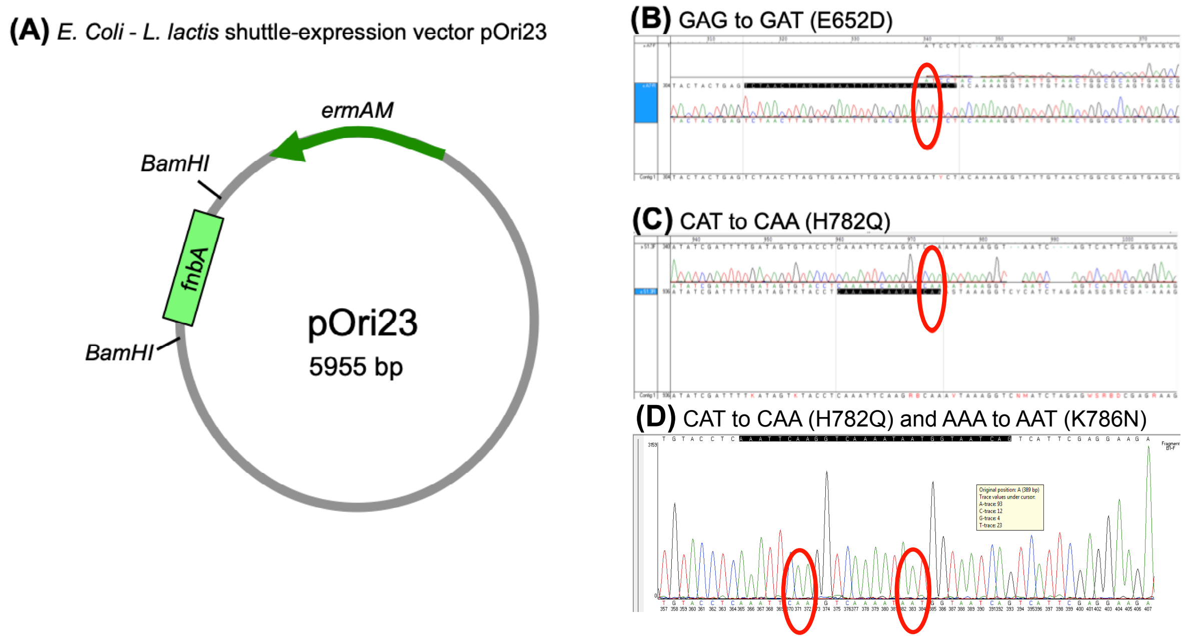

Supplementary Figure S1. (a) Schematic representation of the *E.coli - L. lactis* shuttle-expression vector pOri23 with the *fnbA* gene of *S. aureus* 8325-4 ligated at the BamHI sites and also carrying the *ermAM* macrolide-lincosamide-streptogramin resistance gene. (b-d) Representative chromatograms of the fragment of *S. aureus fnbA* gene showing desired mutations. The sequences were analyzed using Vector NTI after site directed mutagenesis and the desired change in nucleotide sequence was conformed for all seven FnBPA variants.

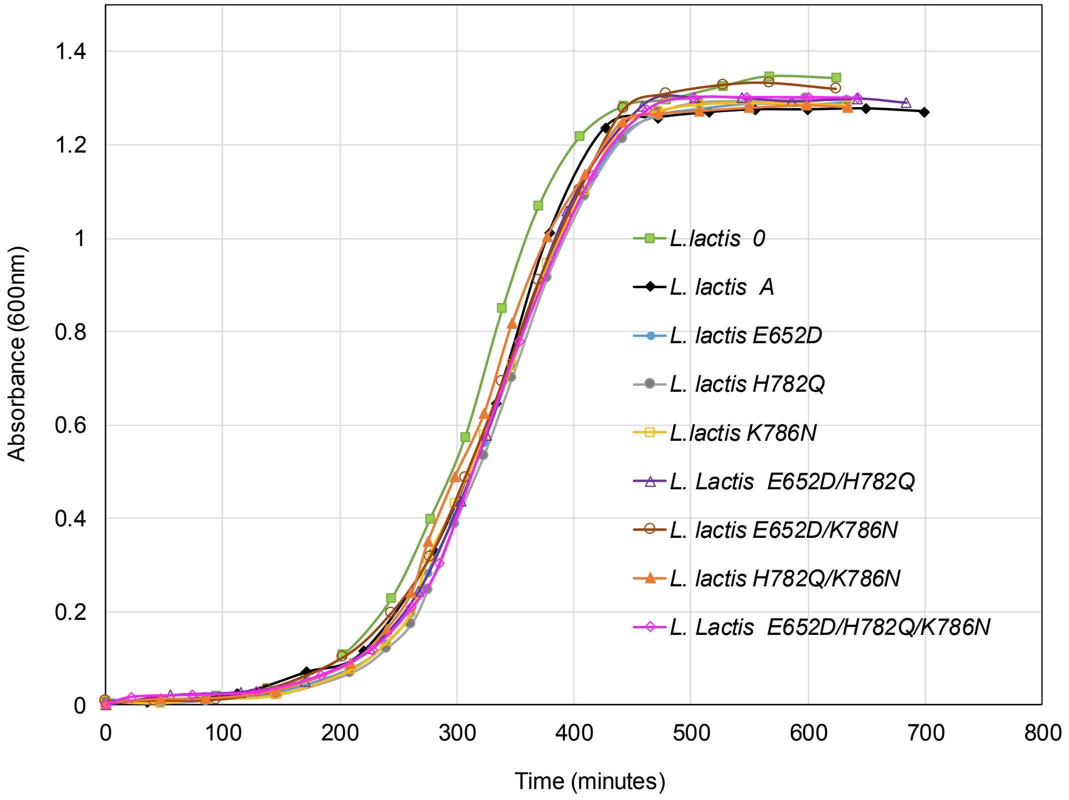

Supplementary Figure S2. Growth curves for the various *L. lactis* strains used in this study.

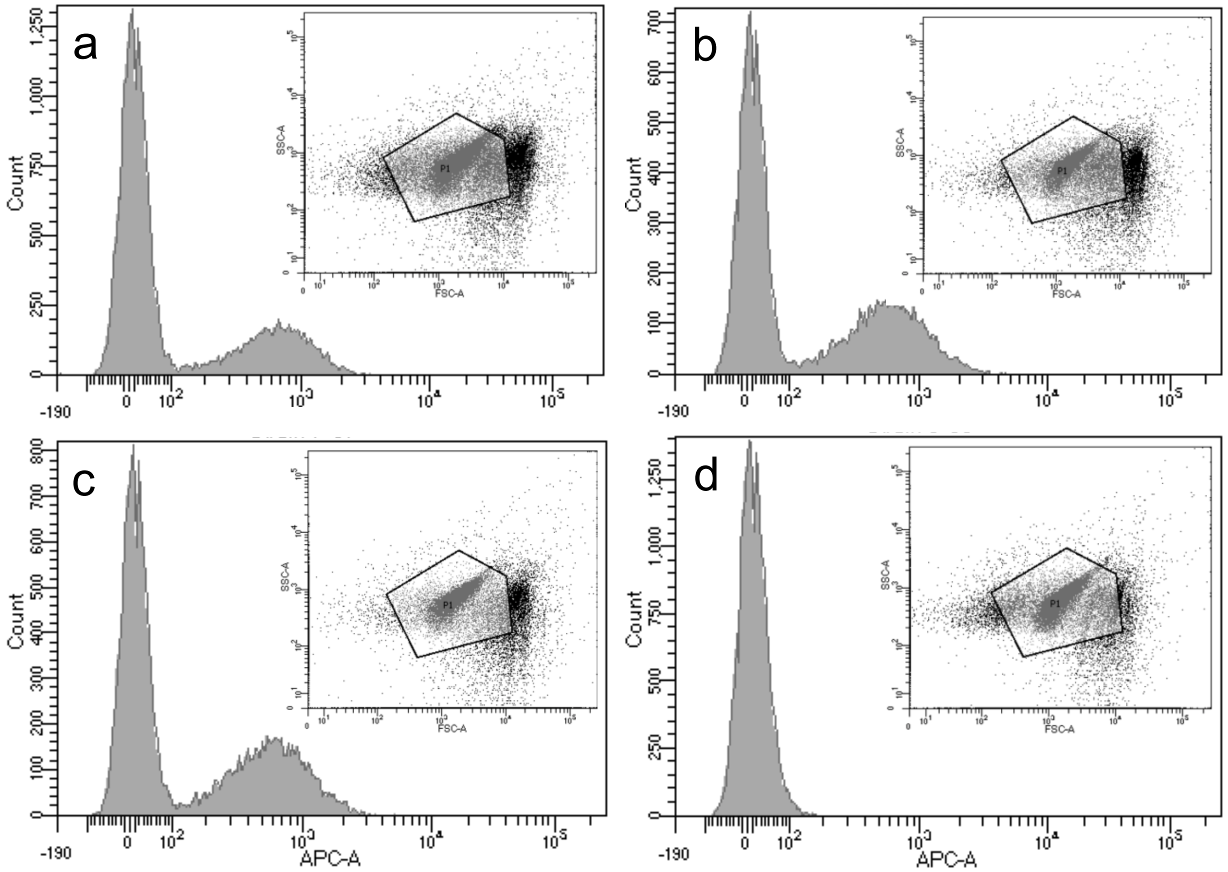

Supplementary Figure S3. FACS flow cytometry analysis of FnBPA expression in *L. lactis* strains carrying wild-type FnBPA (a), E652D variant (b), E652D/H782Q/K786N variant (c), and the empty vector (*fnbA* gene absent) (d). Unstained controls (cells not expressing FnBPA) are shown in the peak on the left, whereas the peak on the right is assigned to the cell population expressing FnBPA (absent in d). Cells were incubated first with a primary antibody (α-FnbpA -polyclonal rabbit sera) and then a fluorescent-dye conjugated second antibody (Goat Anti-Rabbit Alexa 647). A total of 30,000 events were collected per sample. All the *L. lactis* cells express comparable amounts of FnBPA based on the analysis of the peak area ratios (FnBPA variants average 1.6±0.5, and wild type FnBPA 2.7±0.1). Note the different scales of the y-axes.

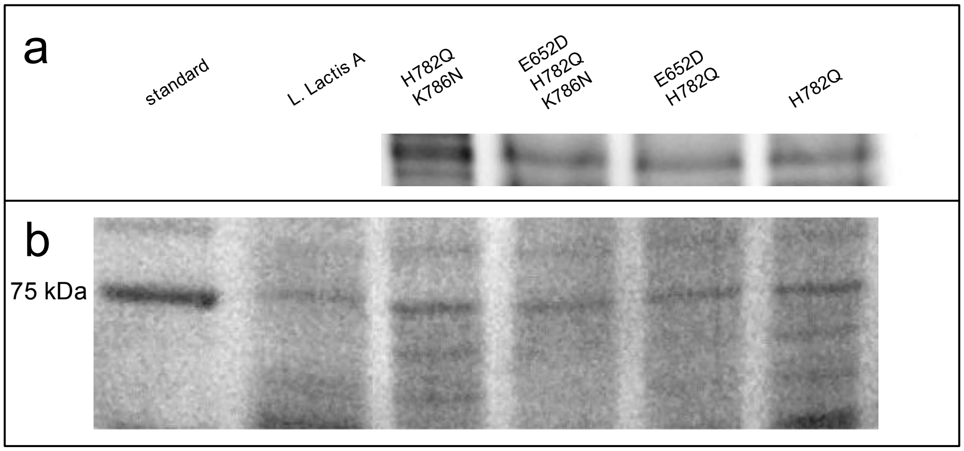

Supplementary Figure S4. (a) Western blots of selected cells wall extracts of *L. lactis* carrying the shuttle-expression vector pOri23-*fnbA* and its mutants (H782Q/K786N, E52D/H782Q/K786N, E652D/H782Q, H782Q). These FnBPA variants were detected by ligand affinity blotting by incubation with pure human Fn and monoclonal antibody against the N-terminus of Fn. A band of ca. 70 kDa that corresponds to a protein fragment of human FN I is observed in all the *fnbA* carrying samples. The average band intensity in the fragment shown for the *fnbA* mutants is 104±4% relative to that of the wild-type *fnbA*. Ponceau *S stain*-loading control (b) for each sample shows similar quantities of total protein were loaded in the gels. The average band intensity in the fragment shown for the *fnbA* mutants is 112±10% relative to wild-type *fnbA*. These two panels have been cropped for clarity. Full-length gel/blots are shown below.

Full-length image of Supplementary Fig. S4, panel a. Green box highlights the region of interest shown above.

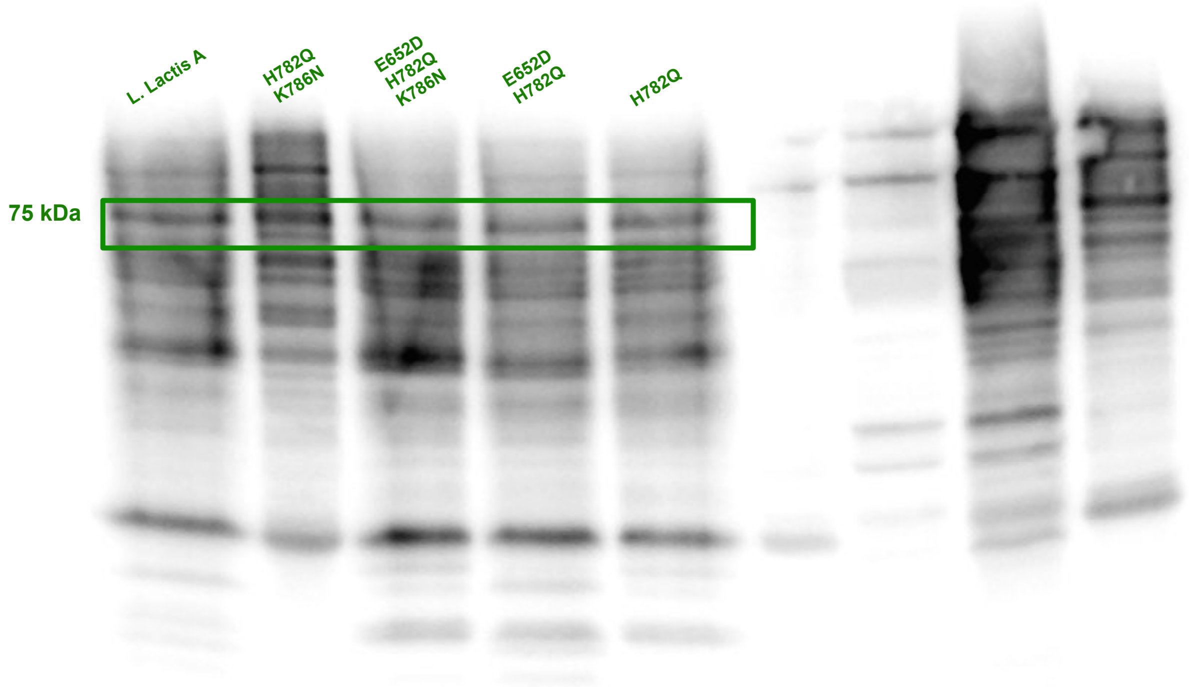

Full-length image of Supplementary Fig. S4, panel b. Blue box highlights the region of interest shown above.

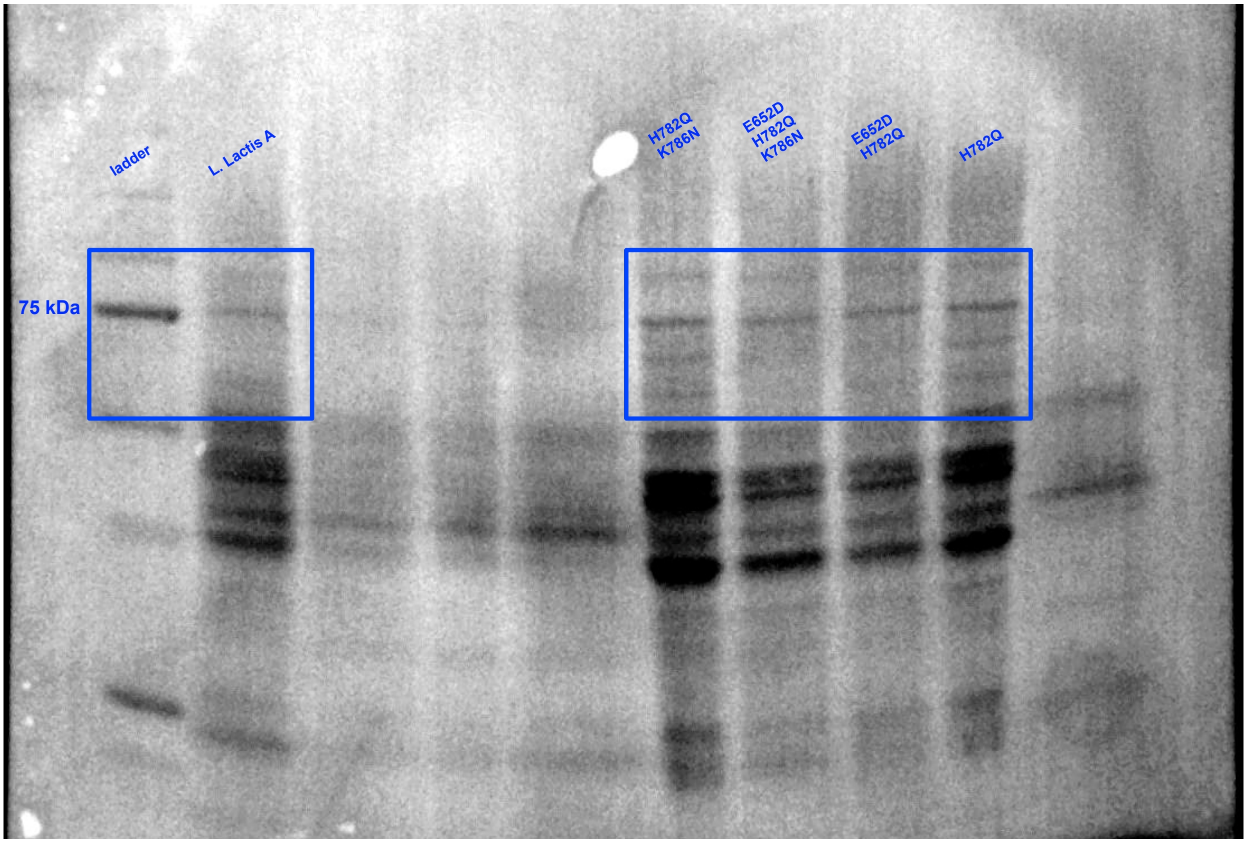

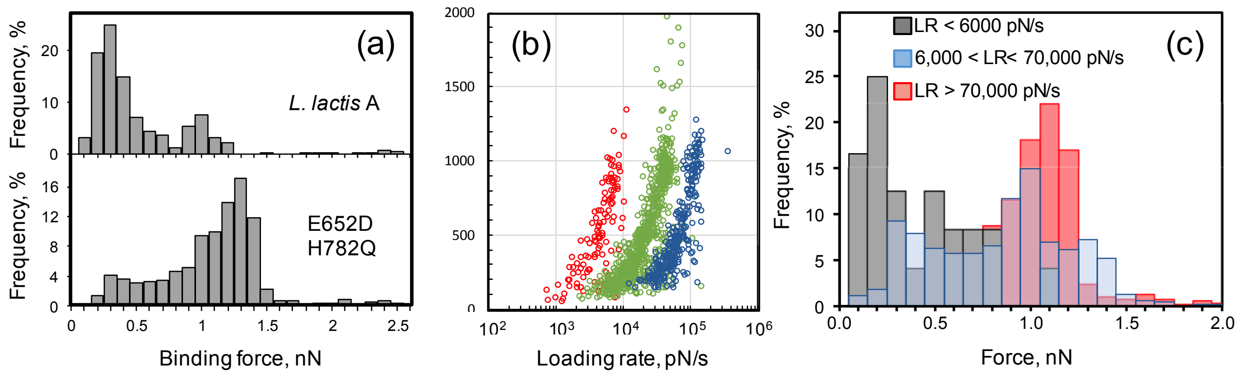

Supplementary Figure S5. (a) Histogram representation of the binding forces of FnBPA expressed in *L lactis* and Fg attached to the AFM tips through N-hydroxysuccinimide (NHS) surface chemistry. One group of binding forces at ~200 pN is observed in both strains. A second set of higher binding forces (e.g., see ~1.3 nN) is present only in the E652D/H782Q variant. (b) Dependence of binding or dissociation force (*f*; pN) on the logarithm of the loading rate, (*r*; pN s^{-1}) between Fg and FnBPA expressed on the surface of the *L. lactis* E652D/H782Q. Different force-loading regimes (shown as different colors) are indicative of multiple binding pathways. (c) Force distributions of the Fg-FnBPA interactions over different ranges of loading rates (LR). The probability of forming strong bonds increases with the rate of the load. LR values were binned <6,000 pN/s (gray bars), from 6,000 to 70,000 pN/s (blue bars), and >70,000 pN/s (red). AFM measurements were conducted in PBS at retraction velocities from 0.05 to 18.8 μm/s. Prior to force measurements, AFM tips were functionalized with Fg through NHS linkage chemistry (see Methods section in manuscript for details on this grafting protocol).

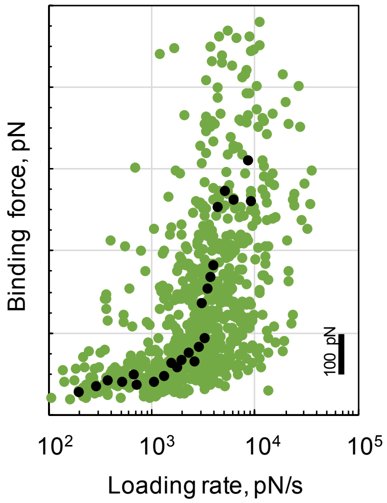

Supplementary Figure S6. Dependence of binding or dissociation force (*f*; pN) on the logarithm of the loading rate, (*r*; pN s^{-1}) between FnBPA expressed on the surface of the *L. lactis* E652D/H782Q and Fn. Raw data are shown in green colored data points, whereas the binned data (the most probable rupture force within a range of loading rates values) are shown in black. The binned data are shown in Figure 5b in the manuscript.
